# AKT抑制剂联合芦可替尼对CALR基因突变骨髓增殖性肿瘤小鼠的治疗作用

**DOI:** 10.3760/cma.j.cn121090-20240920-00360

**Published:** 2025-08

**Authors:** 丽伟 张, 启岗 张, 梦楚 季, 昆明 齐, 振宇 李, 开林 徐, 春玲 付

**Affiliations:** 徐州医科大学血液病研究所，徐州医科大学附属医院血液科，徐州 221000 Department of Hematology, Institute of Hematology, Xuzhou Medical University, Affiliated Hospital of Xuzhou Medical University, Xuzhou 221000, China

**Keywords:** 骨髓增殖性肿瘤, 芦可替尼, AKT抑制剂, CALR基因突变, Myeloproliferative tumor, Ruxolitinib, AKT inhibitor, CALR gene mutation

## Abstract

**目的:**

探讨AKT抑制剂MK2206联合芦可替尼在CALR基因突变的骨髓增殖性肿瘤中的作用。

**方法:**

①获取小鼠骨髓c-kit^+^细胞：颈椎脱臼处死小鼠，分离股骨、胫骨及髂骨，收集骨髓细胞，分选出c-kit^+^细胞。②构建CALR移植小鼠模型：利用逆转录病毒体系构建GFP标记的CALR基因突变载体，经Platinum-E细胞包装后，感染小鼠的骨髓c-kit^+^细胞，经尾静脉注入经致死剂量照射的同种雌性受鼠体内。③移植成功后随机分成四组，分别予以Captisol（15％）、MK2206（60 mg/kg）、芦可替尼（30 mg/kg）、MK2206（60 mg/kg）联合芦可替尼（30 mg/kg）灌胃治疗，连续给药4周，每周给药5 d，监测小鼠外周血血象变化，观察移植小鼠脾脏大小重量。④流式细胞术检测脾脏及骨髓组织中GFP^+^肿瘤细胞、巨核谱系细胞以及造血干细胞比例，观察小鼠脾脏以及骨髓组织肿瘤细胞浸润程度。

**结果:**

①在灌胃治疗后，MK2206联合芦可替尼治疗组的小鼠外周血血小板和白细胞计数均显著低于MK2206单药治疗组和芦可替尼单药组（*P*值均<0.05）；②在移植后第30周，MK2206联合芦可替尼治疗组小鼠脾脏重量低于MK2206单药和芦可替尼单药组（*P*值均<0.05）；③连续给药4周后，联合给药组CALR移植小鼠骨髓以及脾脏组织中巨核谱系细胞、GFP^+^肿瘤细胞比例明显降低（*P*值均<0.05）；骨髓组织中异常造血干细胞占比明显下降（*P*<0.05）；④联合给药组小鼠脾脏及骨髓中肿瘤细胞浸润减轻、异常巨核细胞比例降低。

**结论:**

AKT抑制剂MK2206联合芦可替尼可显著改善CALR基因突变骨髓增殖性肿瘤小鼠的疾病症状与肿瘤细胞浸润。

## Abstract (en)

**Objective:**

To investigate the combined therapeutic role of the AKT inhibitor MK2206 and Ruxolitinib in treating Myeloproliferative Neoplasms（MPN）driven by a calreticulin（CALR）gene mutation.

**Methods:**

① Murine bone marrow c-kit^+^ cells were isolated by sacrificing mice and harvesting bone marrow from the femur, tibia, and ilium for subsequent c-kit^+^ cell sorting. ② A CALR transplantation mouse model was established. GFP-tagged retroviral vectors containing either the CALR gene mutation or the migR1 control were constructed, packaged in Platinum-E cells, and used to transduce murine bone marrow c-kit^+^ cells. These transduced cells were then transplanted into lethally irradiated female recipient mice via tail vein injection. ③ Following successful engraftment, the mice were randomly assigned to four treatment groups for intragastric administration. Complete blood counts were monitored periodically, and the spleen size and weight of transplanted mice were measured. ④ Flow cytometry was used to quantify the proportions of GFP^+^ tumor cells, megakaryocytic lineage cells, and hematopoietic stem cells in both splenic and bone marrow tissues. Histopathological examination was performed to evaluate the degree of tumor cell infiltration in these organs.

**Results:**

① Following gavage treatment, peripheral blood platelet（PLT）and white blood cell counts were significantly lower in the combined AKT inhibitor MK2206 and Ruxolitinib group compared to the MK2206, Ruxolitinib, and control groups（*P*<0.05）. ② In comparison with the MK2206 and Ruxolitinib monotherapy groups, the combination therapy group exhibited a significant reduction in spleen weight and a marked improvement in splenomegaly at 30 weeks post-transplantation（*P*<0.05）. ③ After four weeks of continuous treatment, combined administration resulted in a significant decrease in the proportion of megakaryocytic lineage cells and GFP^+^ tumor cells in the bone marrow and spleen（*P*<0.05）. Additionally, the proportion of hematopoietic stem cells in the bone marrow was also significantly reduced（*P*<0.05）. ④ Histopathological analysis（H&E staining）of bone marrow and spleen tissues confirmed that the combined regimen decreased both tumor cell infiltration and the proportion of abnormal megakaryocytes in these organs.

**Conclusion:**

The combination of AKT inhibitor MK2206 and Ruxolitinib is effective at significantly ameliorating disease symptoms and reducing tumor infiltration in vivo in mice with a myeloproliferative tumor transplantation driven by a CALR gene mutation.

钙网蛋白CALR是一类骨骼肌肌质网膜上的钙结合蛋白。C-末端区域呈强酸性，有很高的钙结合容量，募集钙离子并参与内质网内外钙离子的运输，调节细胞稳态。CALR基因突变是继Janus激酶2或血小板生成素受体（JAK2/MPL）突变后，近年来在骨髓增殖性肿瘤（MPN）中发现的新驱动基因突变。迄今为止，在MPN中已经发现了近50种不同的CALR突变体[1]。所有类型的突变都非常一致地导致1个碱基对的读框移位，导致C-末端氨基酸由酸性突变成中性，同时产生共同缺乏内质网保留序列KDEL的新型C-末端的蛋白，失去钙结合及运输功能[2]–[3]。突变后的CALR常与细胞膜上的受体如MPL结合，过度活化胞内信号通路，从而诱发MPN的发生[4]。

CALR突变检出率在原发性血小板增多症（ET）患者中为20％～25％，在原发性骨髓纤维化（PMF）患者中为20％～30％。在JAK2与MPL突变阴性的ET与PMF患者中，CALR突变检出率高达80％～85％，提示CALR突变在MPN发病过程中发挥了重要作用，尤其是在JAK2与MPL突变阴性的MPN患者中[5]。

目前，JAK1/JAK2抑制剂芦可替尼仍是FDA批准的首个针对高危型MPN/PMF的靶向药物。芦可替尼在改善症状负荷、缓解脾脏肿大和降低细胞因子风暴方面具有显著疗效，但大多数患者在2～3年内进展或不能耐受[6]–[7]，且不能减少基因突变负荷和逆转骨髓纤维化[8]–[9]。我们前期研究发现，AKT在CALR基因突变的细胞中被过度激活，应用AKT抑制剂可减少CALR基因突变小鼠的脾脏重量及异常巨核细胞的数目，联合应用AKT抑制剂与芦可替尼可对CALR突变细胞产生协同抑制作用[10]，提示AKT抑制剂与芦可替尼的药物联合有望成为治疗CALR基因突变类型MPN的新策略。因此，本研究利用CALR基因突变移植小鼠模型，从体内水平深入探索AKT抑制剂MK2206联合芦可替尼对CALR基因突变的MPN小鼠的治疗效果。

## 材料与方法

一、实验动物

本研究经徐州医科大学实验动物伦理委员会批准。实验所用雌性C57BL/6J小鼠均为6～8周龄，由维通利华实验动物技术有限公司提供，于徐州医科大学血液科动物饲养间恒温屏障系统中饲养。

二、主要试剂和仪器

带有GFP标签的MigR1及MigR1-CALRdel52的逆转录病毒载体由美国西北大学Crispino教授赠送；Platinum-E（Plat-E）细胞为本实验室冻存；MK2206购于TargetMOI公司；胎牛血清购于Gibco公司；OPTI-MEM购于Gibco公司；StemSpan™ SFEM培养基购自STEMCELL公司；hLDL、hIL-6、m-IL3以及mSCF均购于美国PeproTech公司；CD117磁珠分选柱购于MACS公司；antimouse c-kit-PE、antimouse Lineage-V450均购自美国BD公司；anti-mouse Sca-1-APC、anti mouse CD41-PE-Cy7购于美国Biolegend公司；40 µm细胞滤网和100 µm细胞滤网为JET BIOFIL公司产品；FACS Calibur流式细胞仪（美国BD公司）；倒置显微镜Ⅸ71（日本OLYMPUS公司）；血细胞计数仪（深圳迈瑞生物医疗公司）。

三、实验方法

1. 构建携带CALRdel52突变基因的小鼠原代细胞：Plat-E细胞按1.5×10^6^/孔的密度铺板，促转染试剂轻轻混匀，EP管中加入无血清OPTI-MEM、2 µg目的质粒，按照1∶3的比例加入促转染试剂6 µl，混匀后室温孵育15 min，将转染复合物滴至Plat-E细胞培养皿中，48 h和72 h收集病毒上清。

颈椎脱臼处死小鼠，75％乙醇消毒腹部皮肤，剪开腹部皮肤，钝性分离股骨、胫骨、髂骨，置于预冷的Facs-buffer中。将所分离的鼠骨置于无菌组织研磨器中轻轻压断，吸取适量Facs-buffer，冲出骨髓细胞，并用100 µm细胞滤网过滤，重复操作3～4次，全部收集于离心管中，×393 *g*离心5 min，获得单细胞悬液。

离心结束后与CD117阳性磁珠共同避光孵育10～15 min，洗去未结合的磁珠，将分选柱置于分离器磁场中。先用40 µm的细胞滤网过滤细胞悬液，防止分选柱堵塞，将骨髓细胞悬液加入分选柱后，取适量Facs-buffer洗涤分选柱，获得磁性标记的骨髓细胞。×393 *g*离心5 min收集细胞，用含有细胞因子的造血干细胞培养基重悬细胞，于37 °C、5％CO_2_细胞培养箱中培养过夜。

离心机预热至32 °C，将孔板中的培养基吸出，留500 µl左右，按照1∶1比例加入500 µl病毒原液，按照1∶1 000加入polybrene，充分混匀后，32 °C、×1 455 g离心90 min，离心结束后，放入培养箱1 h。重复感染一次。

2. 小鼠移植：以γ射线照射C57BL/6J小鼠（7.5 Gy），经尾静脉注入MigR1及MigR1-CALRdel52逆转录病毒转染后的C57BL/6小鼠c-kit^+^细胞。移植后每周检测小鼠全血细胞计数（CBC），13周后移植小鼠出现白细胞增多和血小板增多。

移植后26周，将小鼠随机分成四组，分别予以Captisol（15％）、MK2206（60 mg/kg）、芦可替尼（30 mg/kg）、MK2206（60 mg/kg）联合芦可替尼（30 mg/kg）灌胃治疗，连续给药4周，每周给药5 d。监测给药后移植小鼠的白细胞、血小板指标。

3. 移植后小鼠脾脏和骨髓流式分析及HE染色：在移植后第32周处死小鼠，用流式细胞术和组织化学染色法（HE）检测脾脏和骨髓中肿瘤细胞的浸润情况，监测GFP^+^肿瘤细胞浸润情况以及白细胞、血小板计数等指标。

取小鼠的脾脏以及骨髓研磨，经过细胞滤网过滤，获取单细胞悬液，并用红细胞裂解缓冲液裂解，收集细胞并用PBS洗涤，置于离心管内备用。骨髓造血干细胞（Sca-1-PE-Cy7）、c-kit（c-kit-APC）和巨核谱系细胞（CD41-PE-Cy7）检测：在流式上机管中，分别加入按照1∶100加入相应抗体各1 µl、细胞悬液50 µl、PBS50 µl，4 °C避光孵育30 min，PBS洗涤1次，393×*g*离心5 min，用200 µl PBS重悬细胞，上流式细胞仪检测，并使用FlowJo软件分析数据。

无菌解剖分离小鼠脾脏和股骨胫骨，置于包埋盒中，用4％甲醛固定液固定。常规脱水、浸蜡、包埋后，显微镜下观察肿瘤细胞的浸润情况并进行HE染色检测分析。

四、统计学处理

采用SPSS21.0和GraphPadPrism8.0进行统计学分析和绘图，符合正态分布的计量资料用“均数±标准差（*x*±*s*）”表示；两组间比较若方差齐，采用*t*检验，方差不齐采用Mann-Whitney非参数检验，*P*<0.05表示差异有统计学意义。

## 结果

一、构建CALR基因突变的MPN/PMF移植小鼠

本研究中所用到的CALR突变载体为CALR基因9号外显子缺失52个碱基的缺失型突变载体。该突变基因构建到逆转录病毒载体MigR1上。我们将带有GFP标签的MigR1-CALRdel52突变和MigR1空载体质粒分别转染Plat-E细胞，48 h观察Plat-E细胞的荧光表达强度，并通过流式细胞术检测GFP^+^细胞比例，与WT型Plat-E细胞相比，经过MigR1或CALRdel52转染的Plat-E细胞GFP阳性率分别达到60.4％和61％（图1A）。从雌性C57BL/6J小鼠中收集鼠c-kit^+^细胞，并通过CD117磁珠纯化。用新鲜的MigR1或CALRdel52逆转录病毒悬液感染所获取的c-kit^+^细胞，24 h通过流式细胞术检测GFP^+^细胞比例分别为23.4％和32.9％（图1A），将转染的细胞移植到经7.5Gy照射的C57BL/6J雌鼠体内，构建CALRdel52基因突变的小鼠原代模型。移植第13周后通过尾静脉采血，持续监测CALRdel52基因突变的移植小鼠血常规情况以及外周血中GFP^+^细胞占比，确认CALRdel52突变的MPN小鼠模型构建成功。移植第26周，血小板计数升至1 500×10^9^/L，处于疾病进展期，此时将小鼠随机分为4组，给予灌胃治疗，治疗4周后将小鼠处死后取材分析（图1B）。

**图1:**
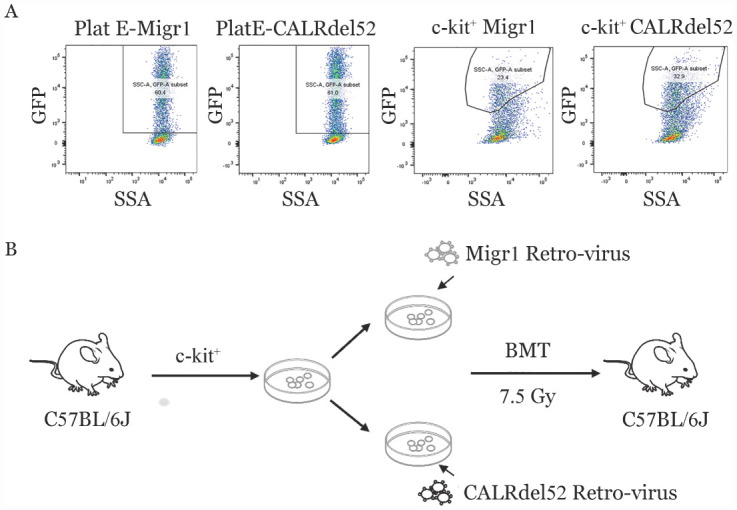
构建CALRdel52突变的骨髓增殖性肿瘤（MPN）小鼠模型 **A** MigR1和MigR1-CALRdel52逆转录病毒感染造血干细胞24 h后GFP^+^细胞流式细胞术分析；**B** 小鼠骨髓移植示意图

二、AKT抑制剂MK2206联合芦可替尼对CALRdel52移植小鼠外周血血象及脾肿大的影响

我们前期研究已经明确，AKT抑制剂联合芦可替尼对CALRdel52基因突变的小鼠原代细胞具有协同抑制作用[10]。因此，为了明确二者是否在CALRdel52小鼠疾病模型中发挥同样作用，我们首先利用逆转录病毒体系构建CALRdel52基因突变的小鼠原代模型。通过外周血全血球分析检测，移植小鼠在移植后20周，血小板计数上升至1 200×10^9^/L，处于起病初期；移植后26周，血小板计数上升至1 500×10^9^/L，处于疾病中期（图2A）。此时，给予AKT抑制剂MK2206（60 mg/kg）联合芦可替尼（30 mg/kg）治疗，连续给药4周。给药期间，通过外周血全血球分析检测发现，对照溶剂组小鼠血小板计数从1 500×10^9^/L逐渐升至2 400×10^9^/L，30 mg/kg芦可替尼单药组治疗后移植小鼠血小板计数从1 500×10^9^/L升至1 800×10^9^/L，减缓了血小板数目增加的速度，而60 mg/kg MK2206处理组血小板数目并没有改善（图2A）。同样，在给予CALRdel52突变小鼠30 mg/kg芦可替尼单药治疗后，WBC从（35～40）×10^9^/L降低至（22～30）×10^9^/L，但仍没有恢复到正常水平。60 mg/kg MK2206使WBC从（34～39）×10^9^/L降至（32～34）×10^9^/L（图2B）。值得注意的是，当MK2206联合芦可替尼处理移植小鼠可使PLT的数目均从疾病中期水平（>1 500×10^9^/L）回落到起病初期（1 200×10^9^/L），WBC基本回落到野生小鼠水平［（18～22）×10^9^/L］（图2A、B）。无论在单药还是联合处理组中，治疗前后各组的HCT水平未有明显改变，维持在55％（图2C）。治疗结束后，剥离小鼠脾脏发现，MK2206或芦可替尼单药组并未起到较好的缩脾效果，其脾脏重量的中位数分别为0.12（0.10～0.13）g、0.11（0.10～0.12）g，而联合给药组小鼠的脾脏重量中位数为0.10（0.08～0.11）g，对照组脾脏重量中位数为0.12（0.11～0.13）g。经Shapiro-Wilk和Kolmogorov-Smirnov检验符合正态分布，分析发现联合组小鼠脾脏平均重量低于对照组小鼠（0.096 g对0.118 g，*P*＝0.008）。

**图2:**
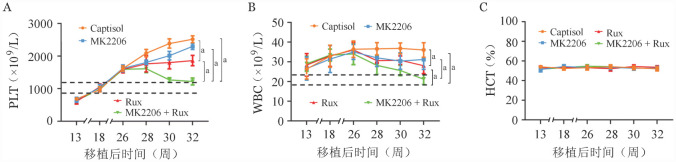
AKT激酶抑制剂联合芦可替尼治疗后CALRdel52突变骨髓增殖性肿瘤（MPN）移植小鼠外周血细胞计数及脾脏的变化 **A** 外周血小板计数；**B** 外周血白细胞计数；**C** 外周血红细胞压积（HCT）；**D** 脾脏重量（所有实验独立重复3次，^a^*P*<0.05）

三、AKT抑制剂MK2206联合芦可替尼对CALRdel52移植小鼠脾脏和骨髓异常巨核谱系细胞的影响

为了进一步评估MK2206和芦可替尼联合作用的效果，在药物治疗结束后，取小鼠的脾脏骨髓进行流式细胞术分析。我们发现，MK2206联合芦可替尼治疗组小鼠的脾脏和骨髓中GFP^+^肿瘤细胞比例明显下降，均显著低于芦可替尼以及MK2206单药组，分别为17.7％，16.3％（图3）。而MK2206或芦可替尼单药组脾脏和骨髓中GFP^+^肿瘤细胞比例并没有明显变化，GFP^+^肿瘤细胞比例平均维持在20％（图4A、B）。此外，在CALRdel52移植小鼠脾脏组织中MK2206或芦可替尼单药组并未起到降低异常巨核谱系细胞比例的作用，联合治疗组小鼠脾脏的异常巨核谱系细胞比例显著降低，降至6.86％（图4C）。不同于脾脏组织中的是，与对照组相较，30 mg/kg芦可替尼单药组可降低CALRdel52突变小鼠骨髓中异常巨核谱系细胞比例，60 mg/kg MK2206单药组并没有起到抑制作用，MK2206联合芦可替尼治疗组小鼠骨髓的异常巨核谱系细胞比例均显著低于芦可替尼以及MK2206单药组，异常巨核谱系细胞比例降低至4.07％（图4D）。

**图3:**
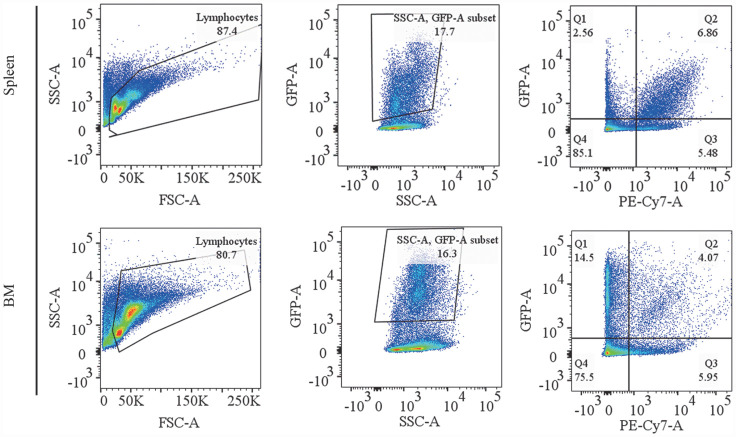
脾脏和骨髓中GFP^+^肿瘤细胞和巨核谱系细胞百分比的流式细胞术分析

**图4:**
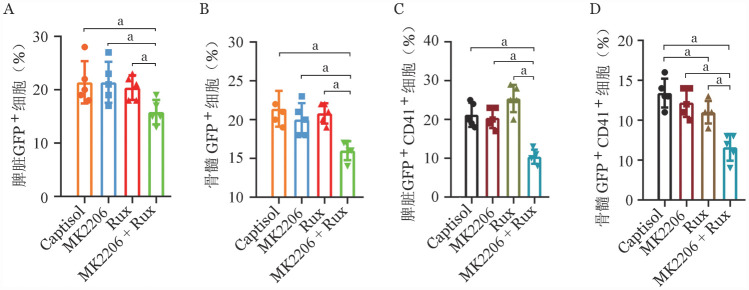
脾脏和骨髓中GFP^+^肿瘤细胞和巨核谱系细胞百分比（所有实验独立重复3次，^a^*P*<0.05）

四、AKT抑制剂MK2206联合芦可替尼对CALRdel52移植小鼠脾脏和骨髓异常造血干细胞的影响

在治疗结束后，取CALRdel52移植小鼠的脾脏骨髓进行流式细胞术分析，发现相对于对照组，MK2206或芦可替尼单药组以及联合给药组小鼠的脾脏组织中异常造血干细胞比例并无明显改变，维持在2％～3％，各药物治疗组与对照组之间并无统计学差异（图5A、B）。但在骨髓组织中，MK2206联合芦可替尼治疗组小鼠的骨髓中异常造血干细胞均显著低于单药组，低于5％（图5C）。而MK2206或芦可替尼单药未起到改善异常造血干细胞的作用，与15％ Captisol组相比无明显差异（图5D）。

**图5:**
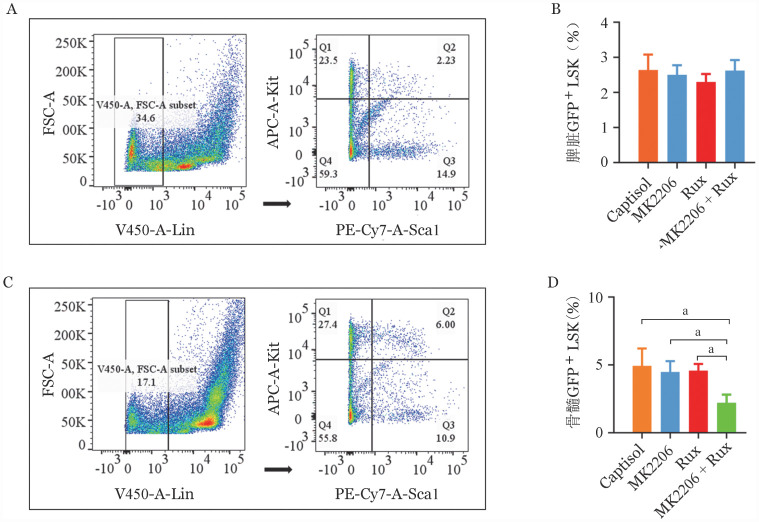
AKT激酶抑制剂联合芦可替尼对脾脏和骨髓组织中肿瘤干细胞比例的影响 **A、B** 骨髓组织中肿瘤干细胞流式细胞术分析及比例；**C、D** 脾脏组织中肿瘤干细胞流式细胞术分析以及比例（所有实验独立重复3次，^a^*P*<0.05）

五、AKT抑制剂MK2206联合芦可替尼对CALRdel52移植小鼠脾脏和骨髓浸润情况

治疗4周后，取小鼠脾脏和骨髓HE染色发现，芦可替尼以及MK2206单药组小鼠的脾脏中肿瘤细胞浸润减少，但未观察到脾脏结构的损伤有明显改善。而MK2206联合芦可替尼治疗组小鼠的脾脏红髓和白髓区域界限清晰，肿瘤细胞的浸润明显降低，脾脏结构有所改善（图6A）。与Migr1组小鼠相比，15％Captisol组骨髓中可见胞体大，分叶多的巨核细胞散在分布，而各治疗组骨髓中异型巨核细胞比例明显下降（图6B）。

**图6:**
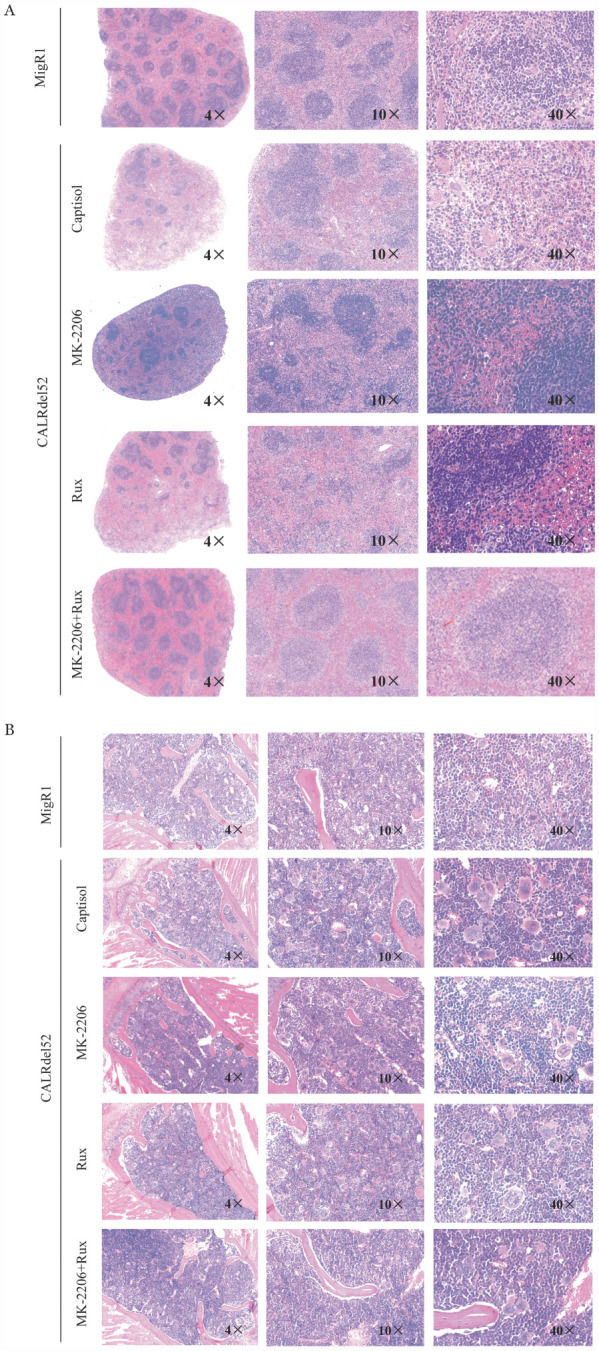
AKT激酶抑制剂联合芦可替尼对CALRdel52移植小鼠脾脏（A）和骨髓（B）肿瘤细胞浸润的影响

## 讨论

JAK-STAT信号通路过度激活在MPN发病机制中发挥了核心作用[11]，是促使第一个JAK1/2抑制剂芦可替尼的开发的重要因素，该抑制剂是改善许多MF患者症状和提高生活质量的最佳治疗药物。虽然JAK2抑制剂已显示出临床症状的改善和明显的生存获益，但骨髓抑制、贫血和血小板减少是该药物治疗下最常见的不良反应[12]–[14]，此外尚未显示出阻止疾病转化风险的能力[15]–[16]，仍是芦可替尼临床疗效不足的重要体现。与其他药物联合治疗MPN，降低芦可替尼的使用量，以提升疗效、减轻不良反应，是未来治疗MPN的优选策略。已有研究显示，全脱乙酰酶抑制剂Panobinostat与芦可替尼具有协同作用，在MF的2期研究中，一些患者的脾肿大、骨髓纤维化和JAK2V617F等位基因负担减少[17]。此外AuroraA激酶抑制剂Ⅰ期临床试验显示，AuroraA激酶抑制剂Alisertib能够促使异常巨核细胞正常化，有效缓解了29％和32％的患者的脾肿大和症状负担，减轻骨髓纤维化[18]。有研究发现，AKT是MPN中CALR基因突变类型疾病进展的重要因子[19]，联用AKT抑制剂与芦可替尼对CALR突变细胞产生显著的协同抑制作用[15]，提示该治疗方法有望成为治疗该类型MPN的潜在策略。

本研究发现，二者联合应用与同等剂量单药相比，具有显著改善CALRdel52移植小鼠白细胞升高及脾肿大的症状，这与联合应用AKT抑制剂与芦可替尼改善MPLW515L突变类型的MPN小鼠疾病进展结果相一致[15]。此外，二者联合应用显著改善了CALRdel52移植小鼠血小板增高的症状，这是高剂量AKT抑制剂单药所无法解决的问题[10]。先前研究结果显示，120 mg/kg的AKT抑制剂对脾肿大及脾脏中肿瘤细胞的抑制作用较为显著，对于改善血小板的水平具有一定的效果[10]。Dutta等[20]和Rampal等[21]的研究显示，在MPLW515L和JAK2V617F基因敲入的MPN小鼠给予芦可替尼单药或联合其他药物治疗，60 mg/kg芦可替尼能够降低肿瘤负荷。而本研究中，60 mg/kg的AKT抑制剂联合30 mg/kg芦可替尼可显著纠正CALRdel52移植小鼠血小板升高及缓解脾肿大。可见，二者联合应用极大减少了药物的起效剂量。此外，无论是单药组或是联合给药组，对CALRdel52移植小鼠的红细胞压积并未产生显著影响，这与我们先前高剂量应用AKT抑制剂的现象相一致[10]。也有研究显示25 mg/kg MK2206可缓解JAK2V617F突变MPN小鼠红细胞增高的症状，但仍然没有使其恢复到正常水平[22]。此外，我们的研究还发现，二者联合应用对CALRdel52基因突变小鼠中骨髓的肿瘤细胞杀伤作用较脾脏显著，表现为可显著减少肿瘤造血干细胞以及其衍生出的异常巨核谱系细胞。同时，病理结果也同样显示，二者联合应用一定程度上改善了脾脏中肿瘤细胞浸润的程度，修复了脾脏的红髓及白髓分区，并减少骨髓中幼稚巨核细胞的数目。

综上，本研究结果显示，AKT抑制剂MK2206联合芦可替尼对CALRdel52基因突变的MPN小鼠具有较好的协同治疗作用，这为其进一步在MPN患者临床治疗应用提供重要的研究基础。
